# Prenatal Dexamethasone for Congenital Adrenal Hyperplasia: An Ethics Canary in the Modern Medical Mine

**DOI:** 10.1007/s11673-012-9384-9

**Published:** 2012-07-31

**Authors:** Alice Dreger, Ellen K. Feder, Anne Tamar-Mattis

**Affiliations:** 1 Clinical Medical Humanities and Bioethics, Program in Medical Humanities and Bioethics, Feinberg School of Medicine, Northwestern University, 750 N Lake Shore Dr., Suite 625, Chicago, IL 60611 USA; 2 Department of Philosophy and Religion, American University, Washington, DC USA; 3 Advocates for Informed Choice, Cotati, CA USA

**Keywords:** Congenital adrenal hyperplasia, Dexamethasone, Medical ethics, Gender identity, Sexual orientation, Human subjects research

## Abstract

Following extensive examination of published and unpublished materials, we provide a history of the use of dexamethasone in pregnant women at risk of carrying a female fetus affected by congenital adrenal hyperplasia (CAH). This intervention has been aimed at preventing development of ambiguous genitalia, the urogenital sinus, tomboyism, and lesbianism. We map out ethical problems in this history, including: misleading promotion to physicians and CAH-affected families; de facto experimentation without the necessary protections of approved research; troubling parallels to the history of prenatal use of diethylstilbestrol (DES); and the use of medicine and public monies to attempt prevention of benign behavioral sex variations. Critical attention is directed at recent investigations by the U.S. Food and Drug Administration (FDA) and Office of Human Research Protections (OHRP); we argue that the weak and unsupported conclusions of these investigations indicate major gaps in the systems meant to protect subjects of high-risk medical research.

## Introduction/Our Backgrounds

In this article, we provide a condensed history of the use of prenatal dexamethasone for congenital adrenal hyperplasia, with an eye toward the ethically problematic aspects of this history. Congenital adrenal hyperplasia (CAH) is a disease of the endocrine system that can cause virilization (i.e., development of masculine traits) in female fetuses. In an attempt to prevent CAH-affected female fetuses from developing in a sexually atypical fashion, some physicians treat pregnant women “at risk” for having an affected daughter with the steroid dexamethasone. This intervention starts as soon as pregnancy is confirmed and continues throughout the pregnancy if the fetus is ultimately diagnosed as a CAH-affected female. If—several weeks into the dosing—the fetus is determined to be male or not CAH-affected, the intervention is immediately stopped, because the intention is only to alter the course of development in CAH-affected females.

This use of dexamethasone was first described in 1984 in *The Journal of Pediatrics* by Michel David and Maguelone Forest, French clinician-researchers. David and Forest reported apparent effectiveness of prenatal dexamethasone in eliminating genital virilization in a single girl affected by CAH (David and Forest 1984). In the nearly three decades since David and Forest’s paper, many specialists have come to believe that prenatal dexamethasone for CAH constitutes the standard of care. A 2000–2001 survey of members of the European Society for Paediatric Endocrinology, representing 125 institutions, found that, “[i]n 57 % of the centres prenatal diagnosis and treatment [of CAH with dexamethasone] are routine” (Riepe et al. 2002, 199). A 2010 Continuing Medical Education review article in the *Obstetrical and Gynecological Survey* concluded: “Given the data available at this moment, antenatal treatment with corticosteroids is recommended” (Vos and Bruinse 2010, 203).

But what data are “available at this moment” such that prenatal dexamethasone for CAH can be recommended to obstetricians, and recommended by them to prospective patients?

A systematic review and meta-analysis of this intervention, published in 2010 in *Clinical Endocrinology*, indicated that a search of the literature “identified 1083 candidate studies for review; of which, only four studies were confirmed eligible” for serious scientific consideration (Fernández-Balsells et al. 2010, 438). That is to say, as late as 2010, less than one half of one percent of published “studies” of this intervention were regarded as being of high enough quality to provide meaningful data for a meta-analysis. Even these four studies were of low quality:

All the eligible studies were observational and were conducted by two groups of investigators (one from the US and one from Europe). … Studies lacked details regarding the use of methodological features that protect against bias. None of the studies reported blinding of the outcome assessors to the exposure (i.e., the researchers estimating each patient’s degree of virilization). Loss to follow-up was, in most cases, substantial (Fernández-Balsells et al. 2010, 438).

In spite of at least a thousand pregnant women likely having been exposed by the time of the review, the four studies judged worthy of inclusion in the meta-analysis covered only 325 pregnancies. Even more stunning, the meta-analysis revealed, “*there were no data on long-term follow-up of physical and metabolic outcomes in children exposed to dexamethasone*” prenatally for CAH (Fernández-Balsells et al. 2010, 436, *emphasis added*). It was not that the data about long-term physical and metabolic outcomes were unclear; there simply were none available.

Today, some clinicians promote prenatal dexamethasone for CAH as “an excellent example of pharmacological therapy during pregnancy” (Rosner et al. 2006, 803) and even as “a paradigm of prenatal diagnosis and treatment” (Nimkarn and New 2010a, 5). Yet the Endocrine Society Task Force that had commissioned the 2010 meta-analysis concluded: “The evidence regarding fetal and maternal sequelae … is of low or very low quality due to methodological limitations and sample sizes” (Speiser et al. 2010a, 4137). This would hardly seem to qualify prenatal dexamethasone for CAH as “an excellent example” or a “paradigm” of a prenatal pharmacological intervention. Indeed, for lack of quality clinical studies, the 2010 Task Force could not even say with any confidence whether prenatal dexamethasone works to reduce genital virilization. Notice the specific qualifications included in the Task Force’s statement on efficacy: “*[T]he groups advocating and performing prenatal treatment appear to agree* that it is effective in reducing and often eliminating virilization of female fetal genitalia and that the success rate is about 80–85 %” (Speiser et al. 2010a, 4138, *emphasis added*).

Regardless of some people’s enthusiastic endorsements of the intervention, we show below that ethical debates within medicine about prenatal dexamethasone for CAH are actually not new. Those internal debates, however, have focused on potential risks and benefits to mothers and children exposed. There are a number of other ethical problems in the history of this intervention also deserving of attention, including: de facto experimentation on fetuses and pregnant women, largely outside of prospective long-term trials and without adequate informed consent; failure to appropriately collect and publish evidence when promoting and providing a high-risk intervention; use of medicine and public monies for research to prevent benign behavioral sex variations, including tomboyism and lesbianism (cf. Murphy 1997); and inadequacy in the United States of systems designed to protect subjects of medical experimentation, including especially pregnant women and their offspring.

In the United States, the use of prenatal dexamethasone remains “off-label.” This means that the indication has never received approval by the Food and Drug Administration (FDA).^1^ Nonetheless, we want to be clear: The problem we see with the use of prenatal dexamethasone for CAH is not per se that it is an off-label use; it is rather that—as this paper documents—prenatal dexamethasone for CAH has sometimes been promoted to prospective patients and clinicians in misleading ways, and sometimes promoted for uses that are not legitimately medical (e.g., for the prevention of tomboyism and lesbianism). Furthermore, this intervention—*intended* to alter the course of fetal development—has been “studied” in ways so slipshod as to breach professional standards of medical ethics.

We come to this work as history, philosophy, and legal scholars interested in the medical treatment of children with atypical sex, but also as women who have long advocated clinical reform in this general area. Late in 2009, clinicians working in the pediatric care of children born with sex anomalies made one of us (Dreger) aware of their growing alarm about prenatal dexamethasone for CAH. These clinicians were concerned that pregnant women at risk for having daughters with CAH were being given prenatal dexamethasone without being informed that (a) this use has consistently been labeled experimental by expert panels; (b) benefits and risks have not been established, due to inadequate scientific study; and (c) some children who had been exposed in utero were being studied retrospectively, in many cases years later, by the very clinicians who had been (and were still) actively promoting the use to pregnant women as “safe for mother and child,” to find out what the risks might really be.

Dreger (a historian) then reviewed the medical literature as well as Internet-based advertisements directed at affected families and became quite concerned. In December 2009 and January 2010, respectively, Dreger and UCLA pediatric geneticist Eric Vilain separately asked Mount Sinai School of Medicine pediatric endocrinologist Maria New, the most prominent promoter of this intervention, about the informed consent process she used. New is a highly distinguished pediatric endocrinologist and member of the National Academy of Sciences. By 2003, she had already publicly taken credit for having “treated” more than 600 pregnant women with dexamethasone in an attempt to prevent virilization in CAH-affected female fetuses (Kitzinger 2003), putting her efforts in this area well beyond any other clinical researcher’s (see also New et al. 2001). Dreger’s preliminary research indicated that, even while obtaining a federal grant promising to determine the actual safety and efficacy of prenatal dexamethasone for CAH through retrospective follow-up studies, New was functioning as a uniquely aggressive promoter of the intervention among parents and clinicians, repeatedly describing this intervention as “safe for mother and child” (New 2010a, ¶4). But when Dreger and Vilain attempted to ask New about informed consent, both were rebuffed by New. Indeed, at the Miami medical conference session where Vilain pressed the issue (at New 2010b), New publicly admonished Vilain that his question to her was inappropriate.

Dreger then asked colleagues in bioethics and allied fields to join with her in raising concerns to the U.S. government regarding the potential failure to protect the rights of these women and their offspring. The second author of this paper (Feder) became the corresponding author on the resulting (February 2010) letters of concern from a total of 32 academicians to the Office of Human Research Protections (OHRP) and the FDA. The third author (Tamar-Mattis) handled most of our subsequent communications with federal agencies and conducted additional legal research.

In September 2010, the OHRP and FDA informed us they could find nothing worth pursuing further. The OHRP decided that abuse had not occurred because: (a) at least some of the pregnant women had been enrolled in IRB-approved studies when given the drug at Weill Cornell Medical College, where most of the interventions appear to have occurred while New worked there; (b) follow-up studies conducted at Mount Sinai School of Medicine under New had been IRB-approved; and (c) the fetal intervention itself does not require IRB oversight. The FDA explained that (to our surprise) regulations allow a clinician to promote an off-label use—even an experimental use intended to alter fetal development—as “safe and effective” so long as the clinician does not simultaneously work for the drug maker or count as an FDA-approved investigator of the drug (Borror 2010). (Dreger has made the agencies’ full responses available at fetaldex.org.) Nevertheless, we show here that the material generated by the government’s own investigations—along with further scholarly inquiry on our part—appear actually to *confirm* the concerns we expressed at the outset, suggesting a major failure of the layered systems designed to protect subjects of research, especially pregnant women and their fetuses.

Because the following history necessarily focuses attention on the actions of Maria New, we want to be sure that our readers appreciate that New’s career has included critically important research and clinical care that has improved the lives, health, and fertility of a very large population. Her promotion of prenatal dexamethasone for CAH was no doubt motivated by a desire to improve the lives of her patients. The same beneficent attitude was likely present in all or most of the clinicians who used prenatal dexamethasone for CAH. But it is worth remembering that many cases in the history of medicine now rightly understood as ethically problematic were carried out by clinical researchers with good intentions (Eder 2011; Reverby 2009; Skloot 2010).

It has been impossible for us not to be aware that, as we have researched this fetal intervention, the medical world has been marking the 40^th^ anniversary of the 1971 publication of a study reporting a relatively high number of occurrences of a rare vaginal cancer in girls and young women who had been exposed in utero to diethylstilbestrol (DES). It was this small 1971 study—eight subjects with adenocarcinoma of the vagina matched to 32 untreated controls—that marked the beginning of the end of DES administration to pregnant women (Herbst, Ulfelder, and Poskanzer 1971). DES treatment during pregnancy had had admirable intentions, including primarily the prevention of miscarriage. Already by 1953, DES had actually been found ineffective for miscarriage prevention, but doctors continued to prescribe it to pregnant women anyway. Over the years, millions of fetuses were exposed, putting females and males at increased risk for rare cancers, genital anomalies, reproductive problems, etc. (Goodman, Schorge, and Greene 2011).

As we have spoken with others about how the use of prenatal dexamethasone for CAH has played out, it has been our experience that many have been skeptical that a DES-like scenario could occur with prenatal dexamethasone for CAH after what physicians learned from DES. We find ourselves wondering whether it is the assumption that “we’ll never do *that* again” that paradoxically has blinded many clinicians to the striking parallels between DES and prenatal dexamethasone for CAH: Both DES and dexamethasone are powerful synthetic hormones; in both cases the practice involved administration of the intervention starting early in pregnancy; both were introduced into medical practice without much study as to efficacy and safety (Dreger et al. forthcoming).

Three striking differences between these interventions are that: (1) this poorly studied yet widespread use of prenatal dexamethasone is happening *after* the lessons supposedly learned from DES; (2) while DES was never intended to alter fetal development, prenatal dexamethasone for CAH has explicitly aimed to do so; and (3) while DES was aimed at preventing fetal death, dexamethasone is directed at preventing something we would hope most people would understand to be substantially less dire, namely the development of atypical sex.

Yet rather than suggesting that the case of prenatal dexamethasone for CAH should be understood as one of the “big” stories of the history of medicine (like DES), we are suggesting something more disturbing: that this case appears to be representative of problems endemic in modern medicine, problems that threaten the health, lives, and rights of patients who continue to become unwitting subjects of (problematic) medical experimentation. Because so many systems of protection appear to have failed these women and children, we fear that prenatal dexamethasone for CAH is a canary in the modern medical mine.

## CAH’s Effects and the Goals of Using Prenatal Dexamethasone

Congenital adrenal hyperplasia (CAH) is a genetic disease involving malfunction of the adrenal glands, endocrine organs that contribute to the production of sex steroids. CAH can occur in both males and females and can cause a number of metabolic problems, some of which may lead to postnatal adrenal crisis and death if left untreated. Because some forms of CAH are very dangerous, all U.S. states require newborn screening for CAH.

The prenatal administration of dexamethasone, a potent synthetic steroid of the glucocorticoid class, cannot prevent an affected child from being born with CAH. The intervention is aimed instead at causing CAH-affected female fetuses to develop in a more female-typical fashion than they otherwise might. Androgens contribute to sex differentiation, including in the brain and genitals; relatively low prenatal levels ordinarily result in a more female-typical development; relatively high levels usually result in male-typical development. In certain forms of CAH—including 21-hydroxylase deficiency (21-OHD CAH), i.e., the type of CAH most at issue here—the prenatal production of high levels of androgens may result in a genetic female (46,XX) fetus developing along a more masculine pathway neurologically and genitally. Prenatal dexamethasone is meant to engineer the CAH-affected female fetus’s hormonal system to be typically female.

The intensity of CAH’s effects on developing females varies. An affected genetic-female child might be born fairly female-typical, or she may be born with a large clitoris, labia that fuse and appear to form a scrotum, and an occluded or even absent lower vagina. Some affected genetic females have developed such masculine genitalia that they have been assumed at birth to be typical males and have been raised as boys (Eder 2011; Lee and Houk 2010). Newborn screening for CAH, aimed primarily at saving lives, has greatly reduced the likelihood of such children’s conditions going undetected.

Atypical clitorises and labia generally require no medical intervention for health. Despite evidence that elective genitoplasty may harm a girl (Crouch et al. 2004), pediatric urologists often perform surgery to “feminize” atypical clitorises and labia for what they call “social reasons,” including promotion of parent–child bonding. They do so even while admitting we lack evidence that this approach is necessary or effective (Lee et al. 2006).

Some CAH-affected girls are also born with a urogenital sinus, a condition in which the urethra and vagina are joined together. This creates potential for repetitive infections and also presents problems for sexual intercourse. Thus the urogenital sinus has traditionally been treated with pediatric surgery. It represents part of what prenatal dexamethasone is meant to prevent.

Women “at risk” of giving birth to a CAH-affected daughter are most often identified because they have already given birth to a CAH-affected child. Others are identified through genetic screening. Because “the period during which the genitalia of a female fetus may become virilized begins only 6 [weeks] after conception, treatment must be instituted as soon as the woman knows she is pregnant” (Speiser et al. 2010a, 4137; cf. Nimkarn and New 2007). Although this intervention is sometimes termed “low-dose therapy” (New 2010c), researchers estimate that “the effective glucocorticoid doses reaching the fetus are 60–100 times physiologic” (Miller 2008, 17), meaning this intervention exposes the developing fetus to 60 to 100 times the normal level of glucocorticoids. The potential harms of prenatal dexamethasone represent a growing source of concern for clinical researchers because emerging research indicates glucocorticoids may alter “fetal programming,” potentially resulting in serious metabolic problems that will not become apparent until adulthood (Hirvikoski et al. 2007; Marciniak et al. 2011). Some animal studies, for example, suggest long-term risk to the cardiovascular system (Lajic, Nordenström, and Hirvikoski 2011).

CAH is an autosomal recessive disorder, so offspring of carrier parents have a 1 in 4 chance of having CAH and thus only a chance of 1 in 8 of being a CAH-affected female (since only half of the 1 in 4 will be females). While recent advances now may enable determination of fetal sex by the seventh week (Devaney et al. 2011), before 2011 the sex status of fetuses could not be determined reliably before 10 to 12 weeks. As a consequence, about 7 out of 8 (87.5 percent) of the individuals who have been exposed to many weeks of prenatal dexamethasone—at 60 to 100 times normal levels—never even had the condition that was being targeted with the prenatal intervention. These individuals have been “necessarily” exposed to risk in order to try to engineer the development of the 1 in 8 who would be CAH-affected females. Many of the clinicians who have raised ethical concerns about prenatal dexamethasone for CAH have specifically been concerned about the 87.5 percent (those 7 out of 8) exposed to the risk of first-trimester glucocorticoid “therapy” who stood no chance to benefit (e.g., Frias et al. 2001; Hirvikoski et al. 2007; Miller 2008; Speiser et al. 2010a).

Most clinicians who have written about prenatal dexamethasone have spoken of its purpose as the prevention of ambiguous genitalia and the urogenital sinus. (Sometimes they speak of the urogenital sinus as part of “ambiguous genitalia,” and sometimes they speak of these as two sets of concerns.) But interestingly, some clinicians also speak of prenatal dexamethasone’s purpose as the prevention of feminizing genital *surgeries*. So one elective risky intervention (prenatal dexamethasone) has been represented as necessary to prevent another (feminizing genital surgeries) (Nimkarn and New 2010a). Notably, in 2006, a major consensus of the American and European pediatric endocrine groups, known as “the Chicago consensus,” urged a more conservative approach to surgeries for genital anomalies, stating that, for girls, “surgery should only be considered in cases of severe virilization” (Lee et al. 2006, e491). But this recommendation does not appear to have had much (if any) effect on the promotion of prenatal dexamethasone as a preventive to “feminizing” surgery.

Studies have shown that girls with 21-OHD CAH exhibit increased rates of what clinicians call “behavioral masculinization,” i.e., behaviors that are more male-typical. These girls are, on average, more interested in boy-typical play, hobbies, and subjects than non-affected females, less interested in becoming mothers, and more likely to grow up to be lesbian or bisexual (Meyer-Bahlburg 1999; Meyer-Bahlburg et al. 2006). The rate of adult identification as male is significantly higher in this group than in the general population of people with an XX karyotype; clinician-researchers report that about 5 percent of CAH-affected genetic-females may ultimately self-identify as male (Dessens, Slijper, and Drop 2005). For this reason, some clinicians have considered recommending raising highly virilized 46,XX CAH-affected babies as boys (Lee and Houk 2010; cf. Eder 2011).

Although many researchers have hinted that prenatal dexamethasone might be good for preventing the “behavioral masculinization” associated with CAH (e.g., Lajic et al. 1998), this potential “benefit” has been spelled out most explicitly in the work of Maria New (Dreger, Feder, and Tamar-Mattis 2010). Writing with another pediatric endocrinologist in the 2010 *Annals of the New York Academy of Sciences* in an article that disregarded the 2006 Chicago consensus, New summed up the situation thus:

Without prenatal therapy, masculinization of external genitalia in females is potentially devastating. It carries the risk of wrong sex assignment at birth, difficult reconstructive surgery, and subsequent long-term effects on quality of life. Gender-related behaviors, namely childhood play, peer association, career and leisure time preferences in adolescence and adulthood, maternalism [interest in being a mother], aggression, and sexual orientation become masculinized in 46,XX girls and women with 21HOD deficiency. … Genital sensitivity impairment and difficulties in sexual function in women who underwent genitoplasty early in life have likewise been reported. We anticipate that prenatal dexamethasone therapy will reduce the well-documented behavioral masculinization and difficulties related to reconstructive surgeries (Nimkarn and New 2010a, 9).

Among those advocating prenatal dexamethasone, New seems to have been particularly concerned that CAH-affected girls may fail to grow up to be heterosexual wives and mothers. Speaking to a group of parents of children with CAH in 2001 at a meeting organized by the CARES Foundation, New showed a photo of a girl with ambiguous genitalia and said:

The challenge here is … to see what could be done to restore this baby to the normal female appearance which would be compatible with her parents presenting her as a girl, with her eventually becoming somebody’s wife, and having normal sexual development, and becoming a mother. And she has all the machinery for motherhood, and therefore nothing should stop that, if we can repair her surgically and help her psychologically to continue to grow and develop as a girl (New 2001a).

In a 1999 paper entitled “What Causes Low Rates of Child-Bearing in Congenital Adrenal Hyperplasia?”, New’s chief collaborator in psychoneuroendocrine studies of CAH, Heino Meyer-Bahlburg of Columbia University, noted:

CAH women as a group have a lower interest than controls in getting married and performing the traditional child-care/housewife role. As children, they show an unusually low interest in engaging in maternal play with baby dolls, and their interest in caring for infants, the frequency of daydreams or fantasies of pregnancy and motherhood, or the expressed wish of experiencing pregnancy and having children of their own appear to be relatively low in all age groups. (Meyer-Bahlburg 1999, 1845–1846).

Meyer-Bahlburg posited that “[l]ong term follow-up studies of the behavioral outcome will show whether [prenatal] dexamethasone treatment also prevents the effects of prenatal androgens on brain and behavior” (1999, 1846).

Surprisingly, results from our Freedom of Information Act (FOIA) requests—made as part of our attempt to understand this history—indicate that the U.S. National Institutes of Health (NIH) have funded New to see whether prenatal dexamethasone “works” to make more CAH-affected girls straight and interested in having babies. New’s 1996 grant application states that

genital abnormalities and often multiple corrective surgeries needed affect social interaction, self image, romantic and sexual life, and fertility. As a consequence, many of these patients, and the majority of women with the salt-losing variant [of CAH], appear to remain childless and single. Preventive prenatal dexamethasone exposure is expected to improve this situation (New 1996a, 38).

New’s NIH grant application specifically promised to try to determine “the success of DEX in suppressing behavioral masculinization” (New 1996b, 17).

## A Long History of Ineffective Calls for Ethical, Scientifically Rigorous Studies

The 2010 systematic review and meta-analysis of prenatal dexamethasone for CAH (mentioned in our introduction) was commissioned by an Endocrine Society Task Force charged with developing new consensus guidelines for the treatment of CAH. That Task Force was in turn co-sponsored by the American Academy of Pediatrics, the Lawson Wilkins Pediatric Endocrine Society, the European Society for Paediatric Endocrinology, the Society of Pediatric Urology, the European Society of Endocrinology, the CARES Foundation, and the Androgen Excess and PCOS Society. Based on the systematic review and meta-analysis, in its 2010 practice guidelines the Task Force concluded

that prenatal therapy *continue* to be regarded as experimental. … We suggest that prenatal therapy be pursued through protocols approved by Institutional Review Boards [i.e., ethics committees] at centers capable of collecting outcomes data on a sufficiently large number of patients so that risks and benefits of this treatment can be defined more precisely (Speiser et al. 2010a, 4137, *emphasis added*).

The named authors of this 2010 consensus (including Meyer-Bahlburg) appeared to have recognized that their call for use only within scientifically-meaningful clinical trials pre-approved by ethics committees was not novel. In fact, expert panels have consistently judged prenatal dexamethasone for CAH to be risky and experimental. In 2002, a joint statement from the Lawson Wilkins Pediatric Endocrine Society and the European Society for Paediatric Endocrinology had already said something similar—even stronger:

We believe that this specialized and demanding therapy should be undertaken by designated teams using nationally or multinationally approved protocols, subject to institutional review boards [IRBs] or ethics committees in recognized centers. Written informed consent must be obtained. … Families and clinicians should be obliged to undertake *prospective* follow-up of prenatally treated children whether they have CAH or not. The data should be entered into a central database *audited by an independent safety committee*. (Joint LWPES/ESPE CAH Working Group 2002, 4049, *emphasis added*).

And a year earlier, in a tense exchange of letters with New in the journal *Pediatrics*, representatives of the Section on Endocrinology and Committee on Genetics of the American Academy of Pediatrics had admonished New that

it remains the physician’s ethical obligation to remain very cautious, even if using lower doses. The fact that only 1 in 8 treated pregnancies may benefit from the therapy confounds this equation even further. There is much information on the effect of glucocorticoids on the brain. Data continue to accumulate that indicate that high-dose glucocorticoid therapy is harmful for the developing (prenatal and postnatal) brain. Further, Dr. New herself coauthored a paper reporting increased frequency of white matter abnormalities and temporal lobe atrophy on magnetic resonance imaging in patients with CAH. Although cause and effect remain to be established, one must consider that glucocorticoid therapy may have played a role in causing these abnormalities (Frias et al. 2001, 805).

The AAP Committee concluded, in their response to New:

The maxim of “first do no harm” requires a cautious, long-term approach, which is why the Academy Committee unanimously agrees that prenatal glucocorticoid therapy for CAH should be confined to centers doing *controlled prospective, long-term studies*. The memory of the tragedies associated with prenatal use of dexamethasone and thalidomide demands no less (Frias et al. 2001, 805, *emphasis added*).

A few months later, *Pediatrics* issued an erratum, correcting what may have been a Freudian slip; the AAP Committee had meant to say “the memory of the tragedies associated with the prenatal use of *diethylstilbestrol (DES)* and thalidomide demands no less” (*Pediatrics* 2001, 1450, *emphasis added*).

Over the years, many individual researchers and clinicians expressed similar concerns about the use of prenatal dexamethasone for CAH outside of appropriate studies or, in some cases, about its use at all (Seckl and Miller 1997; Miller 1999; Ritzen 2001; Hughes 2003; Lajic et al. 2004; Hirvikoski et al. 2007). Even Forest, the French pioneer of the intervention, wrote in 2004, “the prenatal treatment of CAH remains an experimental therapy and, hence, must only be done with fully informed consent in *controlled prospective* trials approved by human experimentation committees at centre’s that see enough of these patients to collect meaningful data” (2004, 479, *emphasis added*). By 2008, Walter Miller, distinguished professor of pediatrics and chief of endocrinology at the University of California–San Francisco, declared, “It is this author’s opinion that this experimental treatment is not warranted and should not be pursued, even in prospective clinical trials” (Miller 2008, 17). Miller later would add: “It seems to me that the main point of prenatal therapy is to allay parental anxiety. In that construct, one must question the ethics of using the fetus as a reagent to treat the parent, especially when the risks are non-trivial” (in Dreger 2010, ¶20).

In spite of all these challenges and warnings, New and her collaborators in the United States appear never to have entered into a prospective, long-term, continuous study of the use of prenatal dexamethasone for CAH. New and her group do appear to have occasionally done some trials of prenatal dexamethasone during pregnancy, tracking outcomes up to the birth; among the 325 pregnancies included in studies seriously considered by the 2010 meta-analysis, 281 came from New’s group (Fernández-Balsells et al. 2010). Even those, however, were not studied in a matter adequate to establish efficacy or safety according to the standards of evidence-based medicine. The trials lacked adequate controls and methods to mitigate bias.

Moreover, our recent FOIA findings on New’s pregnancy trials of dexamethasone throw up multiple ethical red flags. For example, in her 1985 application to the Cornell IRB to study prenatal dexamethasone for CAH, New did not check the boxes indicating that her subject population included pregnant women and fetuses (New 1985, 2), even while, deeper in the application, she indicates that the study design calls for administration of dexamethasone starting at two to four weeks of gestation (New 1985, 9e). The accompanying consent form describes this use as experimental, but then goes on to minimize the risks:

I understand that my participation in the project involves the following risks: transient and reversible suppression of the maternal and fetal adrenal gland. For this reason, extra doses of steroids will be administered to the mother at the time of delivery to cover for the additional stress. Although complications of glucocorticoid therapy (cleft palate, growth retardation, placental degeneration and fetal death) have been reported in laboratory animals, the doses used were extremely high. Congenital malformations associated with dexamethasone therapy are rare in humans, even when large doses are given. The pregnant women and fetuses treated to date with this regimen have not experienced complications (New 1985, 9e).

A pregnant woman reading this might reasonably have read “transient and reversible” to mean that the risks included no long-term harms to her or her offspring.

In another New York Presbyterian Hospital/Cornell Weill Medical College consent form for the prenatal administration of dexamethasone for CAH, marked “IRB Approved” and dated April 2004, New required pregnant women to sign a form saying they understood the use was “experimental.” But the form also advised the women: “Over the last decade, treatment with dexamethasone in the prescribed doses has been shown to be effective in reducing the masculinization of the female fetus with CAH and has been shown to be safe for the fetus” (New 2004).

Thus, it remains unclear—but doubtful—whether U.S. federal regulation 45 CFR 46 subpart B, which regulates experimentation on pregnant women, was consistently followed in this use of prenatal dexamethasone. Because the OHRP removed, from its FOIA response to us, sections of its correspondence with Weill Cornell that might have indicated how many women were included in pregnancy trials of prenatal dexamethasone conducted by New, we have been unable to determine how many of the 600-plus pregnant women and fetuses she says she had “treated” at Weill Cornell by 2003 had the benefit of IRB surveillance (such as it was). What we do know is that, given what we have seen, even those engaged in IRB-approved trials at Weill Cornell apparently cannot be said to have given informed consent.

So far as we can ascertain, there has been and remains an absence of IRB oversight for prenatal dexamethasone administration for CAH at Mount Sinai, where New began working in June 2004, in spite of the fact that New has claimed the exposure of fetuses at Mount Sinai as evidence of her research progress there. For example, in her 2006 grant “progress report” to the NIH, New indicated that she and her team had settled in at Mount Sinai and had been continuing the intervention there: “The prenatal diagnosis and treatment program has galloped ahead so that we now have diagnosed and or treated 768 fetuses,” including 27 fetuses exposed to dexamethasone from February 2005 to January 2006, i.e., when she was at Mount Sinai (New 2006, 3).

Our FOIA digging also turned up a 2004 letter from Jeffrey Silverstein, chair of the Mount Sinai IRB, assuring the NIH that New had Mount Sinai IRB approval for a project called “Prenatal Diagnosis and Treatment” (Silverstein 2004); the accompanying documents indicate that the project specifically involved the use of prenatal dexamethasone for CAH. (New probably needed this IRB approval letter to continue her NIH funding.) Yet in his 2010 response to the OHRP on behalf of Mount Sinai in response to our letters of concern, Silverstein indicated that Mount Sinai did not believe IRB oversight of the decision to undertake this fetal intervention was needed, because New was not herself writing the prescriptions (Silverstein et al. 2010).

Mount Sinai’s 2010 conclusions that New had not been doing research on those pregnancies appears to be based on the confusing premise that *other* doctors had been doing the prescribing to the pregnant women whose fetuses were then included in the growing numbers of New’s subjects. Perhaps Mount Sinai’s respondents to the OHRP in 2010 did not know that New had been reporting statistics on fetal exposure as part of the federally-funded research progress she described to the NIH as “gallop[ing] ahead” at Mount Sinai? But surely Silverstein, who lead-authored the Mount Sinai defense in 2010, should have known that he had himself assured the government in 2004 that New was intending prenatal exposure as part of a study supposedly under IRB supervision. If these fetuses were not regarded as research subjects locally in terms of having IRB protections, they appear to have at least been counted as research subjects federally.

With Meyer-Bahlburg, New has also conducted a few follow-up studies, mostly low-quality questionnaire studies in which parents have been asked, long-distance, to reply to a battery of questions. (The 2010 review found these of such low quality, they did not even bother to consider them for the meta-analysis, and Meyer-Bahlburg has admitted that “fewer than 50 % of mothers and offspring have responded to questionnaires” [Speiser et al. 2010b, under “3. Prenatal Treatment of CAH”].) For these studies, the researchers had IRB approval, but it does not appear that the families enrolled in these studies would have known that they might have been misled about the status of prenatal dexamethasone when it was administered to the pregnant women. Thus it is questionable whether their participation in the follow-up study can be called fully informed.

So far as we can ascertain, New was not alone in her less-than-rigorous approach to scientific study of prenatal dexamethasone for CAH. Indeed, although the intervention has been offered in many other nations, Sweden appears to be the only nation to have specifically restricted prenatal dexamethasone for CAH to women who agreed to participate in a continuous, prospective, long-term study of the intervention, a restriction instituted there in 1999. (Before that it could be obtained outside of trials.) The group has been led by Svetlana Lajic, associate professor of molecular medicine and surgery at the Karolinska University Hospital. In a recent exchange with one of us (Feder), Lajic revealed that the Swedish team has actually halted the intervention part of the study due to concerns about adverse effects.

Lajic wrote to Feder that “due to our findings we have addressed the Regional Ethics Committee in Stockholm, in November 2010, and stated that due to possible adverse events we wish to put on hold further recruitment of patients to the on-going prospective study of prenatal DEX treatment of CAH” (Lajic 2011). While our paper was in press, the Swedish team published news of this development, along with follow-up data on “43 children treated in Sweden and Norway during 1985–1995” (Hirvikoski et al. 2012, 1). When compared to controls,

[i]n general, treated children were born at term and were not small for gestational age. As a group, they did not exhibit teratogenous effects/gross malformations, although eight severe adverse events were noted in the treated group, compared with one in the control group. Three children failed to thrive during the first year of life; in addition, one had developmental delay and hypospadias; one had hydrocephalus; two girls were born small for gestational age, and one of these girls was later diagnosed with mental retardation; and one child had severe mood fluctuations that caused hospital admission. In the control group, only one child was admitted because of Down’s syndrome (Hirvikoski et al. 2012, 2).

The Swedish team has clearly been alarmed by the extraordinary number of serious medical problems among the small group of children treated prenatally.

As for the cognitive and behavioral outcomes, in their most recent report the Swedish team acknowledged that “The small sample size, relatively high refusal rate, and the retrospective study design limited the conclusiveness of the results” of their follow-up of the behavioral development in 40 Swedish children treated prenatally. Nevertheless, they could report that

[a]n adverse effect was observed in the form of impaired verbal working memory in CAH-unaffected short-term-treated cases [i.e., the children who were not the intended targets of the intervention]. The verbal working memory capacity correlated with the children’s self-perception of difficulties in scholastic ability, another measure showing significantly lower results in CAH-unaffected, DEX-exposed children. These children also reported increased social anxiety. In the studies on gender role behavior, we found indications of more neutral behaviors in DEX-exposed boys (Hirvikoski et al. 2012, 2).

In other words, the boys appeared relatively less masculine—another unintended effect.

The Swedish team argued that “the[se] results cause concern because no side effects should be tolerated in CAH-unaffected children who do not benefit from the treatment per se” (Hirvikoski et al. 2012, 2). Indeed, the team concluded its 2012 report with as strongly a worded ethics statement as has ever been issued by a team engaged in the use of prenatal dexamethasone for CAH: “We find it unacceptable that, globally, fetuses at risk for CAH are still treated prenatally with DEX without follow-up” (Hirvikoski et al. 2012, 2).

As we urged United States governmental agencies in 2010, the Swedish team now “urge[s] the scientific community to perform additional retrospective studies, preferably on all treated children and young adults” (Hirvikoski et al. 2012, 2). Yet we see no signs that rigorous, independently-audited, retrospective studies will be pursued in the United States, where the great majority of interventions appear to have occurred, largely under the guidance of Maria New. Although we find plenty of suggestions in New’s grant materials and presentations that results from her retrospective follow-up studies are forthcoming, and although the NIH repeatedly renewed her prenatal dexamethasone research funding, little appears to have been produced in terms of longer-term data on prenatal dexamethasone for CAH, particularly with regard to long-term safety. We can find no publications resulting from New’s 2007 Rare Diseases Clinical Research Network (RDCRN/NIH) follow-up study protocol, except a paper she co-authored in a 2011 issue of *Advances in Experimental Medicine and Biology*. This paper purports to demonstrate the long-term safety of prenatal dexamethasone for CAH. But the article contains no methods section, no description of the study allegedly being reported, no description of the controls, nor even of the intervention. It consists simply of unexplained tables of data and unsupported narrative (New and Parsa 2011). Needless to say, it is not the kind of report that will ever make it into a meta-analysis.

Although New had told the U.S. government in 1996 “we propose to continue our studies of prenatal diagnosis and treatment” (New 1996b, 61), we can find no evidence of there ever having been, in the United States, a reasonably-designed, IRB-approved, prospective, long-term study—nothing like the rigorous approach taken in Sweden. In a recent debate with Dreger on this subject, Meyer-Bahlburg confirmed the total absence of prospective continuous studies in the United States and offered no indication that any has ever even been planned (Meyer-Bahlburg 2011). This is very concerning, and is even more worrisome when one considers the design of New’s 2007 RDCRN/NIH retrospective follow-up study (to be carried out with Meyer-Bahlburg). As we discovered from our FOIA requests, that protocol specifically states as an “exclusion criteria for all groups” this: “mental impairment which prevents understanding of questionnaire” (New 2007, 25).

So, while the researchers in Sweden are documenting cognitive impairment among those exposed to prenatal dexamethasone for CAH, researchers in America have specifically designed a follow-up study that *prevents detection* of certain negative effects on cognitive development.

## Promotion of Prenatal Dexamethasone for CAH

New has a clear track record of promoting the use of prenatal dexamethasone for CAH as safe and effective to clinicians who might reasonably have expected her to represent accurately to them what was actually known and unknown. In a co-authored article for the 2000 edition of the *Cecil Textbook of Medicine*, New advised her colleagues: “Prenatal treatment with dexamethasone has been shown to be safe and effective for both mother and child in the largest human studies” (New and Josso 2000, 1302), implying that there were large human studies of sufficiently high quality to establish safety and efficacy. A few years later, New’s discussion of prenatal dexamethasone (published with two co-authors) in the 2007 edition of the textbook *Pediatric Endocrinology* reads, “we believe that proper prenatal treatment of fetuses at risk for CAH can be considered effective and safe. Long-term studies on the psychological development of patients treated prenatally are currently underway”—a tacit admission of more interest in outcomes related to gender identity and cognitive development than in metabolic outcomes like those involving cardiovascular function (New, Ghizzoni, and Lin-Su 2007, 237).

New’s determined influence on prenatal treatment practices for CAH is also illustrated by the work she has published in *GeneReviews*, the NIH-sponsored free online textbook regarded as an authoritative source in prenatal counseling. Until recently (for reasons we explain below), New’s article described prenatal dexamethasone for CAH as if it were standard practice, explaining precisely how clinicians in the field could implement the intervention. No mention was made of its experimental status, of the fact that the use is off-label, nor of the absence of evidence for its efficacy and safety (Nimkarn and New 2009; cf. Witchel and Miller 2012).

In her grants with the NIH, New also represented prenatal dexamethasone for CAH as having been shown safe and effective. For example, in her 2003 report, she indicated: “Based on our experience and other large human studies, proper prenatal diagnosis and treatment of 21-OHD is safe for mother and child, and is effective” (New 2003a, 98). In her NIH grant materials, New used the fact that “[w]e are the only group in the U.S.A. routinely carrying out prenatal diagnosis and treatment of CAH” as a major reason why the government should fund her studies on the “large population of prenatally-treated infants” she had “accumulated” (New 1996b, 2). Already by her 1996 report to the NIH, New was claiming her clinic “receive[d] requests [for the intervention] from all over the U.S. and foreign countries at the rate of 3–4 per week” (New 1996b, 61). A decade later, New’s protocol for a follow-up study again indicated that her clinic had been drawing patients from all over the United States (New 2007, 25–26); recall that by 2006 she reported use of the intervention in 768 pregnancies (New 2006, 3).

The large draw of potential subjects for her grants perhaps occurred because New actively promoted prenatal dexamethasone for CAH as safe and effective not only to clinicians but also directly to parents, including through the CARES Foundation, a non-profit organization dedicated to supporting individuals and families with CAH and to advancing research and improved clinical practices (e.g., newborn screening). New’s 2001 lecture to parents at CARES, quoted above, represents one example. Another appears in the CARES Foundation Winter 2003 newsletter, for which Elizabeth Kitzinger of Weill Cornell Medical College provided a short article effectively advertising New’s clinic as *the* place to go for at-risk mothers:

In the United States, the only center routinely offering prenatal diagnosis and treatment is Dr. Maria New’s clinic at New York Presbyterian Hospital-Weill Medical Center (Cornell) in New York City. Dr. New has treated over 600 pregnant women at risk for the birth of a CAH-affected child. … The results are remarkable. Dr. New maintains contact with all children treated prenatally, and has found no adverse developmental consequences. Thus, with nearly 20 years’ experience, the treatment appears to be safe for mother and child, though there are endocrinologists who are wary of using dexamethasone prenatally even now (Kitzinger 2003, ¶2–¶3).

Some parents reading this might hear in that last line a warning that not all clinicians were convinced this use was safe and effective. But others might reasonably read it as an indication that Dr. New would be the only clinician willing to help them. The text continues:

It is important to note that prenatal diagnosis and treatment should ONLY be done in a clinic like Dr. New’s with long experience and commitment to follow-up. Only by tracking the growth of prenatally treated children can the long-term effects of treatment be exhaustively studied. Administering dexamethasone to achieve normal genitalia requires the judgment and experience of specialists. The benefits to families of classically affected girls cannot be underestimated. We hope that the availability of this treatment will be shared with all families at risk for the birth of CAH-affected children. (Kitzinger 2003, ¶4, capitalization original).

Even since the 2010 meta-analysis, the website of the Maria New Children’s Hormone Foundation continues to claim “the treatment has been found safe for mother and child” (New 2010a, ¶4). In 2010, our complaints to the FDA about advertisement of this off-label use as “safe for mother and child” were referred to Robert “Skip” Nelson, an FDA-based pediatrician and ethicist. As mentioned above, our discussions with Nelson clarified that regulations prohibit only two groups from advertising off-label uses as “safe and effective”: employees of a drug’s maker (which New is not) and FDA-approved investigators of drugs.

According to Nelson, New did seek and obtain an IND (investigational new drug) exemption from the FDA in 1996 for prenatal dexamethasone for CAH; this would mean she sought and obtained the FDA’s permission at that time for a specific study of this drug use. But as Nelson explained to us, since New does not *currently* have an approval from the FDA to study prenatal dexamethasone, she does not count as an FDA investigator, so she is not currently prohibited from calling this intervention “safe and effective.” In other words, because of a regulatory loophole in the United States, if you inappropriately investigate an off-label use of a drug, then you can also inappropriately advertise it—even to pregnant women and even when the drug use is meant to alter the course of fetal development.

Notably, we think Nelson may be incorrect in his claim that in 1996 the FDA granted New an IND exemption for the use of prenatal dexamethasone to prevent sex atypicality in CAH. Through the FOIA, we obtained the 1996 FDA letter cited by Nelson in 2010, and it actually refers only to a “proposal to utilize dexamethasone to treat pregnant women with a [sic] congenital adrenal hyperplasia” (Sobel 1996). It is entirely possible the 1996 letter referred to a study of the use of dexamethasone to treat women with CAH who became pregnant, i.e., an off-label use meant to treat a woman *herself* afflicted with CAH to keep *her* healthy during her pregnancy*,* an entirely different medical matter than an explicit attempt at fetal engineering wherein the mother is merely a carrier of CAH. There appears to be no evidence to support Nelson’s representation of the 1996 IND exemption letter.

## A Worrisome Track Record

We feel optimistic that our efforts to draw attention to this use of prenatal dexamethasone have increased the likelihood that physicians and researchers will respect the rights of the population exposed. Nevertheless, we feel pessimistic about ever knowing what really happened to most of those already exposed—for weeks or months of pregnancy or fetal development. Recall that in 2002 a joint statement by the European and American pediatric endocrinology societies called for data on prenatal dexamethasone outcomes to be entered into “a central database audited by an independent safety committee” (Joint LWPES/ESPE CAH Working Group 2002, 4049). But—except for a still unpublished European study known as “PREDEX,” which enrolled only 24 subjects from 1999 to 2004, including subjects from the Swedish prospective cohort (Lajic et al. 2004)—no such thing has happened. Instead, it appears from the 2007 RDCRN/NIH study protocol that Maria New retains control of the database of contact information for what she reports is now more than 768 children and their mothers who have been exposed to this prenatal intervention through her clinics (New 2006, 3). It seems likely that that population represents at least a significant minority of those exposed. In fact, New’s 2001 NIH “application for continuation grant” provides a table describing human subjects under the “specific [study] aim” called “prenatal dx and treatment in families at risk,” and there the total number of subjects is stated as 2,144 (New 2001b, 14).

Our research has caused us substantial concern about what appears to be a pattern of misrepresentation by Maria New, even beyond what we take to be misrepresentations regarding the status of prenatal dexamethasone for CAH. As reported in *The Wall Street Journal* in 2005, one of New’s NIH grants (which included work on CAH) formed the subject of a fraud suit brought against Weill Cornell Medical College—a suit over “phantom studies” that resulted in a settlement whereby Cornell paid the government $4.4 million (Wysocki 2005). While the settlement required no admission of wrongdoing, it seems significant that, at the time of the fraud case, New’s status at Cornell radically changed; substantial portions of her Cornell titles and salary appear to have been administratively withdrawn.^2^

An oblique exchange in which we participated in 2010 raises additional concerns about New’s track record for trustworthiness. In a project aimed at making known the experimental status of prenatal dexamethasone for CAH, Dreger wrote with Taylor Sale (Dreger’s student and a genetic counselor) to the editors of *GeneReviews* to request that Dr. New’s article in that publication be changed to reflect medical consensuses concerning prescription of prenatal dexamethasone for CAH (Dreger and Sale 2010). The editors reviewed the consensus statements Dreger and Sale provided them and then wrote to New with recommended changes. Some time later, one of the editors wrote to Dreger and Sale to report that: “Dr. New’s initial reply to our proposed edits to the 21-hydroxylase deficient [sic] CAH *GeneReview* is that ‘Prenatal dexamethasone treatment has been FDA approved by Dr. Sobel.’” The editor added, “We are interested in your thoughts on [Dr. New’s] comment” (Dolan 2010). We informed the editors that Solomon Sobel was the FDA physician who had signed off in 1996 on a single IND exemption (Sobel 1996). As we surely did not need to explain to the *GeneReviews* editors, this did not qualify prenatal dexamethasone for CAH as “FDA approved.”

Today, New’s co-authored *GeneReviews* article on 21-OHD CAH says this: “Prenatal treatment should continue to be considered experimental and should only be used within the context of a formal IRB-approved clinical trial” (Nimkarn and New 2010b, ¶1 under “Therapies Under Investigation”). Since the 2010 revision, the article no longer provides detailed instructions on how to conduct the intervention. And yet, the change reflected in the *GeneReviews* article does not appear to mark a major change in approach for New. Just after the revision of the *GeneReviews* piece, New published a short article in *The American Journal of Bioethics* entitled, “Vindication of Prenatal Diagnosis and Treatment of Congenital Adrenal Hyperplasia with Low-Dose Dexamethasone.” There she wrote: “The recent reports by the Office of Human Research Protections and the FDA … make crystal clear that my research on prenatal treatment of CAH is and always has been both legally and ethically proper at every level” (New 2010c, 68).

In spite of our petitions, the Office of Human Research Protections (OHRP) appears to be quite a bit less concerned than we are about the veracity of New’s claims. For example, the agency appears to have accepted a claim that New had not been conducting fetal experimentation with dexamethasone at Mount Sinai, apparently without learning or considering that her 2006 NIH grant report described “gallop[ing] ahead” with the intervention and specified, as part of her “research progress report,” at least 27 fetuses exposed to prenatal dexamethasone for CAH since New had relocated her clinic to Mount Sinai (New 2006). As part of its investigation following our letters, the OHRP uncovered the deeply problematic IRB-approved consent forms from Weill Cornell mentioned above, yet the OHRP accepted New’s claims that appropriate IRB oversight and informed consent had occurred. The OHRP staff did not indicate in their response to us how many of the pregnant women New claims to have “treated” were even in IRB-approved trials, nor did they address the problematic disjuncture between her own advertisement of prenatal dexamethasone as “safe for mother and child” and her simultaneous federally funded study to investigate whether in fact it is safe for mother and child.

This all seems particularly strange since Tamar-Mattis’s research turned up two strongly-worded 2004 determination letters from the OHRP finding major faults with New’s IRB-approved studies at Weill Cornell (Tamar-Mattis 2010). (OHRP determination letters are the final result of investigations that reveal problems in the conduct of research.) New was not the only researcher at Cornell named in the findings; in fact, the OHRP found the entire system for reviewing pediatric research at Cornell so problematic that they placed a restriction on Cornell’s Federalwide Assurance and took the extraordinary step of requiring review by the IRB of all active research involving children (McNeilly 2004). The problems the OHRP found with protection for subjects in New’s studies were particularly alarming: an informed consent form that did not include a description of the purpose of research, as required (McNeilly 2004, 5); consent forms that contained extremely complex language that a subject would be unlikely to understand (McNeilly 2004, 6); an informed consent form that did not state that “the tests conducted for the protocol could be obtained outside the research” (McNeilly 2004, 5); “subjects enrolled outside the protocol age range prior to IRB review and approval” (McNeilly 2004, 4); missing IRB records (McNeilly 2004, 3) and research initiated without first obtaining legally informed consent (McNeilly 2004, 3). The OHRP also noted that Weill Cornell had suspended New’s research protocol in the fall of 2002 but did not report the suspension to the OHRP until June 2003 (McNeilly 2004, 4).

Why so much scrutiny of New and Weill Cornell by the OHRP in 2004 and so little by the OHRP in 2010? This seems to be part of a problematic trend forming at the OHRP—again suggesting that prenatal dexamethasone for CAH is not a unique case but a bellwether. A March 2011 analysis from the *Report on Research Compliance* on the OHRP has found a substantial drop-off of OHRP determination letters in the last four years: “Agency observers and others expressed concern about the steep drops in letters and open cases, telling [the *Report on Research Compliance*] they raise questions about the agency’s current commitment to serious oversight of human subjects research and investigations into possible wrongdoing” (National Council of University Research Administrators 2011, 1). Notably, the “possibility that compliance is increasing at institutions was not among the reasons OHRP cited for the drop, and funding is not a problem” (National Council of University Research Administrators 2011, 1). Nor has the number of complaints made to the OHRP fallen.

## Final Thoughts

The OHRP and FDA have not yet fully responded to our FOIA requests. (Dreger is presently suing to obtain the remainder of the documents.) But even what we already have found indicates that the government could well have made a case for inappropriate behaviors here and could have issued statements echoing at least some of our concerns about the way that prenatal dexamethasone for CAH has been administered and studied.

Despite the disappointing responses from the OHRP and FDA, we do not regret raising the alarm as we did starting in January 2010. Doing so increased awareness among the affected population about the actual status of prenatal dexamethasone and has also shone light into corners we would not otherwise be able to view. For example, it made available to the public New’s retrospective study protocol and IRB-approved consent forms, and it also led to a reporter for *Time* magazine finding women who indicated that they did not know, when given prenatal dexamethasone to attempt prevention of virilization in female fetuses, that it was an off-label and controversial drug use (Elton 2010). Without the investigation, this valuable information would have remained hidden from view.

The investigation also seems to have driven Mount Sinai to take action, judging from Silverstein’s response to the OHRP:

The Committee [at Mount Sinai charged with responding] determined that there are widely differing opinions amongst the staff, with some staff members expressing significant concerns regarding the use of dexamethasone for the prenatal treatment of CAH. A particular concern is the current necessity to treat potentially unaffected fetuses until a diagnosis is determined. Therefore, the Committee concluded that the clinical use of dexamethasone in this situation should require a rigorous informed consent process with detailed documentation that the risks and benefits of this treatment have been clearly communicated to the parents making a decision to engage in prenatal treatment. The Committee also recommends that this issue be referred to the Medical Board of The Mount Sinai Hospital for further consideration of the consent issue. (Silverstein et al. 2010, 11).

It would appear, then, that Mount Sinai now shares our concerns about the practices associated with the administration of dexamethasone. Nevertheless, it is still the case that the Maria New Children’s Hormone Foundation website declares that prenatal dexamethasone for CAH “has been found safe for mother and child” and provides a phone number to call to make an appointment (New 2010a, ¶4). When the number is called, it rings to a clinic at Mount Sinai.

## Epilogue

As our paper was in press, Maria New’s research group, led by Meyer-Bahlburg, published a study including some cognitive outcome data for 67 children prenatally exposed to dexamethasone for CAH (Meyer-Bahlburg et al. 2012). The children studied came from New’s database (Meyer-Bahlburg et al. 2012, line 92) and included eight CAH-affected girls who were “long-term” exposed in utero and 59 boys and CAH-unaffected girls who were “short-term” exposed (Meyer-Bahlburg et al. 2012, line 23). These children were compared to 73 unexposed controls (Meyer-Bahlburg et al. 2012, line 24).

The new paper concludes: “Our studies do not replicate a previously reported adverse effect of short-term prenatal DEX exposure on working memory, while our findings on cognitive function in CAH girls with long-term DEX exposure contribute to concerns about potentially adverse cognitive aftereffects of such exposure” (Meyer-Bahlburg et al. 2012, lines 34–36). The “previously reported adverse effect of short-term prenatal DEX exposure” was that reported by the Swedish team (Hirvikoski et al. 2007). But whereas the Swedish team employed a relatively rigorous design (a prospective, controlled, long-term study), in the new study from New’s group, the 67 exposed children were selected via convenience sampling performed retrospectively.

Furthermore, although the new Meyer-Bahlburg et al. publication purports to seek information on the effects of dexamethasone exposure (Meyer-Bahlburg et al. 2012, lines 26–27), among the eight girls long-term exposed the degree of exposure varies from a total of nine weeks of fetal life to a total of 39 weeks (Meyer-Bahlburg et al. 2012, lines 117–120), an exposure-length difference of more than four times, making it very difficult to establish meaningful dose-effect.

In marked contrast to the Swedish team, and as if to confirm the weakness of the study by New’s group, Meyer-Bahlburg et al. found some “positive” cognitive outcomes in the short-term treated children. Understandably, they found this hard to explain. (No one suspects first-trimester glucocorticoid exposure at 60 to100 times normal levels to be good for children’s brains.) In their discussion, the authors try to explain the lack of corroboration of their results by other studies or by logic (Meyer-Bahlburg et al. 2012, lines 278–298), but they do not propose the most obvious explanation for this very strange finding: The paper’s study population is a highly skewed sample.

We document here that New has claimed to have “treated” somewhere between 600 and 2,144 fetuses with dexamethasone for CAH, yet her group is now reporting cognitive outcomes on only 67 children in her database, a tiny fraction of those who ought to be available for study. Furthermore, based on New’s claims to the public and to her granting agencies, her efforts should have produced between about 98 and 268 girls who were long-term dexamethasone-exposed, yet here her group reports on cognitive outcomes in only eight. Thus it would appear that the children included in the new study from Meyer-Bahlburg et al. represent somewhere between only 3 and 12 percent of the population prenatally exposed to dexamethasone under New’s consultation.

What the new paper from Meyer-Bahlburg et al. actually appears to replicate is our finding: that the approach taken by New and her collaborators to prenatal dexamethasone for CAH has been so scientifically weak as to be both clinically uninformative and profoundly unethical, especially in light of the history of DES.

## Footnotes

1 For an analysis of the problems with off-label usages, see Dresser and Frader (2009).

2 On February 12, 2003, New wrote to the NIH asking for money to support herself, under the NIH Merit Award system: “As I am no longer Chairman of Pediatrics, Chief of Pediatric Endocrinology, and Program Director of the CCRC, I have much more time to devote to the research proposed in my Merit Award. … This is a hard time for me and I am deeply appreciative of your consideration” (New 2003b). In a subsequent internal memo, the NIH staff “enthusiastically support[ed]” giving New more money under the Merit Award system, without mentioning *why* “her circumstances changed abruptly this year when she had to relinquish the chairmanship of the Department of Pediatrics and the directorship of the Children’s Clinical Research Center at Weill-Cornell Medical School and the salaries entailed in these positions” (NIH Staff 2003). Shortly after, New was hired by Mount Sinai School of Medicine.
